# Indicators for local policies of cultural welfare: content, dimensions, and quality criteria

**DOI:** 10.1186/s40410-022-00179-w

**Published:** 2022-10-17

**Authors:** Annalisa Cicerchia

**Affiliations:** 1 ISTAT - Istituto Nazionale di Statistica, Rome, Italy; 2 Cultural Welfare Center, Turin, Italy

**Keywords:** Arts-and-health, Culture and health, Culture and wellbeing, Cultural indicators, Cultural welfare, Wellbeing indicators

## Abstract

Wellbeing measures are gaining consensus as complementary to the traditional GDP approach when it comes to assessing the development of countries and communities. Cultural Welfare is a new, integrated approach aimed at promoting the wellbeing and health of individuals and communities through practices rooted in the arts and in cultural heritage. Recently, experimental tools have been devised and applied, with reference to either specific target groups of beneficiaries or individual cultural/artistic disciplines, the purpose of which is to measure and assess the contribution to individual and societal wellbeing, as well as the health of cultural and artistic participation and practice in general. Policies aimed at promoting cultural welfare need a robust body of evidence, and indicators may contribute to supporting them. While measures for the assessment of the culture-and-wellbeing relationship have been proposed at the national level or at the level of individual organizations or programmes, the level of local policies appears still largely unexplored. The article discusses a few theoretical and methodological issues and proposes a set of quality criteria for designing cultural welfare indicators on a local scale.

## Introduction

### The long way from GDP to wellbeing: is there a place for the arts and culture?

Simon Kuznets, one of the fathers of the System of National Accounts, and of the concept of GDP, wrote as early as 1934 that “the welfare of a nation can scarcely be inferred from a measure of national income.” (Kuznets 1934). On March 18th, 1968, Senator Robert Kennedy delivered his famous speech on the failure of GDP (it ‘measures everything except that which is worthwhile’)(Kennedy 1968).

Distinguished academic communities in the U.S. were on the same wavelength, and the Social Indicators Movement, advocating the use of social indicators for measuring societal progress and development, was on its way (Noll and Zapf 1994; Land 1983; US Department of Health, Education and Welfare 1969).

From the 1950s to the end of the 1980s, several new measures explored the social preconditions and dimensions of wellbeing as an alternative to GDP-based assessments. Among them, Bauer’s Social Indicators (Bauer 1966), Bhutan’s Gross National Happiness (Royal Government of Bhutan 2012) and Nordhaus and Tobin’s Measure of Economic Welfare (Nordhaus and Tobin 1973), the OECD List of Social Indicators (OECD 1982), and Miringoff’s Index of Social Health (Miringoff 1997) all contributed to the effort. The Brundtland Report (Brundtland 1987) boosted the popularity of the notion of sustainable development, and in 1992, the UN Summit in Rio de Janeiro introduced the same concept into the political debate. Other measures include the UN Human Development Index (UNDP 1994); the Genuine Progress Indicator (Cobb et al. 1995), and the Quality-of-Life Index (Diener and Suh 1997). The issue gained momentum once more during the first decade of the third millennium, also thanks to relevant stirrups such as the Stiglitz-Sen-Fitoussi Report (Stiglitz et al. 2009); the European Commission’s GDP and Beyond projects (Eurostat 2010); OECD’s Better Life Initiative (OECD 2011); the UN General Assembly’s Resolution on Happiness (United Nations General Assembly 2012); and the World Happiness Report (Helliwell et al. 2012). Many of the alternative measures share a domain-based approach: they break down the concept—too big, and too qualitative to be easily and directly measured—into building blocks, or domains. Domains are further deconstructed into themes, themes into eminent phenomena, and phenomena are linked to measurable variables.

Arts and culture are sometimes listed among the key domains which contribute to wellbeing, albeit not frequently. This was the case with two national projects: the Italian Measures of Sustainable and Equitable Wellbeing and the Canadian Index of Wellbeing.

Since 2010, the Italian National Institute of Statistics has been developing a system of Measures of Equitable and Sustainable Wellbeing (Benessere Equo e Sostenibile—BES) that lists Landscape and Cultural heritage among its 12 basic definitional pillars of wellbeing. Moreover, it includes Cultural participation indicators in the domain of Education and indicators related to Occupation in the cultural and creative sector in the domain of Innovation, Research and Creativity (Istat 2013, 2021). The Canadian Index of Wellbeing is built upon eight domains, one of which is devoted to Leisure and culture, with four main components: participation in leisure, recreation, arts, and cultural activities; perceptions/feelings about leisure activities, including why people participate, which needs are being met through participation, and how leisure and culture participation benefits them; Leisure experience, or the meaning it holds for people in relation to their quality of life, and the provision of leisure and culture opportunities, such as access to recreation facilities, open spaces and parks, and other arts, culture, and recreation sites (Canadian Index of Wellbeing 2016). Indexes for measuring wellbeing in cities have been published in recent years (Orii et al. 2020; Pineo and Rydin 2018; Gaffaney 2017; The Wellbeing Project 2017; Chadha et al. 2015). However, only a few of them include culture and the arts as fundamental dimensions of wellbeing. Over the last two decades, scoreboards of indicators have been proposed for measuring *cultural vibrancy or vitality* at the urban level, which are somehow related to quality of life, sustainability, and (indirectly) wellbeing. Among others, the Urban Institute (Jackson et al. 2006); Garcia and Cox (2013) with reference to the European Capitals of Culture—EcoC programme, and the Joint Research Centre with their Cultural and Creative Cities Monitor (Montalto et al. 2019) have all proposed their measures. The United Cities and Local Governments created a system of indicators for assessing cultural sustainability (James 2015). At the end of 2019, UNESCO published Culture Indicators 2030, a system of 22 indicators devised to assess the contribution of culture to the Sustainable Development Goals (SDGs), which are meant for covering both the national and the urban level (Unesco 2019; Cicerchia 2021).

### Arts and health as an emerging topic

Wellbeing and health are closely intertwined. Since 1948, the World Health Organisation has defined health as “a state of complete physical, mental and social wellbeing and not merely the absence of disease or infirmity” (World Health Organization 1948). At the heart of this definition is the recognition of the importance of health promotion, disease prevention, and, above all, the social and cultural determinants of health, i.e., the conditions and abilities needed for people to be well, from both an individual and a social perspective (World Health Organisation 1948, 2008; Allen and Allen 2014). This vision considers health as rooted within society and culture.

The practice of relying on the arts for healing and happiness is ancient (see, among others, Cork 2012; Belfiore 2015; Owen 2013; Sigurdson 2015; Cicerchia 2021). Beauty and harmony contribute to the balance between the body, the mind, and the environment, thus to good health (Clow and Fredhoi 2006; Grossi et al. 2019). At the beginning of the new millennium, an editorial in the British Medical Journal suggested: ‘Spend (slightly) less on health and more on the arts—health would probably be improved’. Doubtless with a hint of provocative intent, the author—a medical doctor and the editor of the authoritative journal—argues that even a minimal diversion of healthcare funds towards the arts would improve the health of people: “The arts don’t solve problems. Books or films may allow you temporarily to forget your pain, but great books or films (…) will ultimately teach you something useful about your pain. […] If health is about adaptation, understanding, and acceptance, then the arts may be more potent than anything that medicine has to offer” (Smith 2002; cited by Belfiore 2015).

Since the 1948 definition, the concept of health has expanded. “Health depend[s] on whether a person has established a state of balance within oneself and with the environment. […] Those with a disease or impairment will be considered as being healthy to a level defined by their ability to […] get the most they can from their life despite the presence of the disease” (Sartorius 2006). On a similar line, the allostasis model defines health as “optimal responsiveness” (Sterling 2019). The *salutogenic* approach does not focus on the elements that threaten health, but rather on those that enable people to manage themselves in the best possible way (Antonovsky 1996). As a relevant source of life skills, “culture, by contributing to the intellectual, emotional, moral and spiritual wellbeing of people, and by enabling everyone to exercise their human rights, including their cultural rights, also contributes to sustainable development” (UNESCO 2015).

In a recent strategic report on the economic and social impact of the cultural and creative sectors, the OECD mentions evidence on how cultural participation positively affects both life expectancy and quality of life, even after statistically controlling for factors such as income, education, or health status (OECD 2021). The comprehensive 2019 World Health Organisation review on the role of the arts in preventing illness and promoting health, as well as in managing and treating illnesses throughout people’s lives (Fancourt and Finn 2019) is specially mentioned; references are made to well-established practices in the arts and health field, such as the “arts on prescription” programmes that recommend cultural experiences (e.g., visiting museums) and cultural and artistic engagement as part of therapeutic plans and active ageing programmes (Dalziel et al. 2019; Clift and Camic 2015; Brandling and House 2009).

Under these premises, the entire range of *behaviour settings* (Lewin 1951; Barker 1968; Gump 1971; Fox and Ghosh 1981; Fox 1985, 1986) where individuals and groups live are essential factors of health and wellbeing for the people involved. To explain human behaviour, we need to look at the environment where this behaviour unfolds. The 2021 American Time Use Survey Well-being Module Questionnaire (US Bureau of Labour Statistics 2021), as a matter of fact, tries to identify the settings of said behaviour. “The arts-and-health field is making an important contribution to the wellbeing agenda in many countries, as the social injustices behind inequalities in health are addressed” (Owen 2013). The resulting strategies are sometimes labeled as *Cultural Welfare*. Cultural Welfare is a new integrated approach to promoting the well-being and health of individuals and communities. Part of this approach is “to include the processes of cultural production and dissemination appropriately and effectively within a welfare system and thus make them an integral part of the social welfare and health services that guarantee citizens the forms of care and support needed to overcome critical issues related to health, ageing, disabilities, social integration and all the problems associated with the recognition of a duty of social protection” (Sacco 2017a, b).

The notion of Cultural Welfare is policy-oriented, and is based on the recognition of the effectiveness of sustained participation in specific cultural, artistic, and creative activities as a factor:

- of subjective well-being, life satisfaction, and health,
- in combating health-related inequalities and fostering social cohesion,
- of active aging, combating abandonment and isolation,
- of inclusion and empowerment for people with disabilities or marginalised people,
- supporting traditional therapeutic practices and the doctor-patient relationship,
- of support for carers, especially non-professional ones,
- of mitigation and delay of several degenerative conditions (Cicerchia et al. 2020).

### Evidence for the policies

In recent years, several experimental tools have been devised and applied with the aim of measuring and assessing the contribution of cultural and artistic participation and practice in general to individual and societal wellbeing and health, also during the Covid-19 pandemic (Hill 2021; Ascolani et al. 2020; Sacco and Grossi 2015; Carnwath and Brown 2014; McLellan et al. 2012; Brajsa-Zganec et al. 2011; Cuyper et al. 2011; Wilkinson et al. 2007; Clow and Fredhoi 2006).

Many of those studies are population-wide, with national and international samples.

Other indicator-based measurements have addressed the impacts of individual artistic disciplines and cultural practices on wellbeing (see, for instance, Clift and Hancox 2010; Clift et al. 2008; Clair 1996 on music; Hui and Stickley 2010; Durdey 2006; Copeland and Cohen 1983 on dance; Clow and Fredhoi 2006 on art galleries; The Beaney House of Art and Knowledge 2021; The Heritage Alliance 2020; Desmarais et al. 2018; Veall et al. 2017; Noble and Chatterjee 2013; Fears 2011 on cultural heritage and museums; Meeks et al. 2020; Michalak 2014; Sextou and Monk 2013 on theatre; Hjort 2019; Cohen et al. 2016; Matthew et al. 2012; Dermer and Hutchings 2010; Powell and Newgent 2010 on cinema).

Other studies report the contribution of the arts and culture to the wellbeing of specific segments of the population with various mental and physical conditions: from the elderly to teenagers, from recent immigrants to newborns, from prison inmates to oncological patients, people with severe depression, Alzheimer’s, or Parkinson’s. A useful open source is the Repository of Arts and health Resources (https://www.artshealthresources.org.uk/repositorysearch/), with over 700 records as of January 2022. The recent report published by the European Office of the WHO offers a scoping review with details on the characteristics of the different beneficiaries (Fancourt and Finn 2019).

Clift and colleagues, however, adopt a more cautious attitude in recalling the early critiques by Belfiore and Mirza (Mirza 2006) on the role of the arts in promoting health and reducing social and health inequalities, and suggest that “broad scoping reviews are ill-advised as a guide for practice and policy development, and future progress should be guided by rigorous, systematic and transparent methods that ensure that review results are trustworthy” (Clift et al. 2021).

Policies aiming at harnessing the full potential of the arts and culture in promoting wellbeing and health need specific, reliable tools to plan interventions and monitor, assess, and evaluate their impacts and results (Cicerchia 2019). Among those tools, besides qualitative in-depth analyses, narratives and stories, scenario techniques, etc., indicators represent a valuable and popular option.

In this exercise, I will discuss a few theoretical and methodological aspects that should be considered when devising indicators aimed at assessing the contribution of culture and the arts to the wellbeing of individuals and groups for the purpose of policy planning and implementation at the local level. I will also propose a first, tentative list of indicators.

## Proposal and discussion

### What we can measure, what we should measure

The indicators we are talking about ideally lie at the intersection of various social and medical sciences, political decision-making systems, and statistics. To be useful, they must strike a balance between policy relevance, scientific consistency, and, of course, measurability. Measurability comes at a price. If indicators, on the one hand, increase the measurability of phenomena, they may lead to their simplification on the other hand. Thus, compared to qualitative information, indicators are more concise and less complex at the same time.

As a result, while improving the quantification, communication, and positioning of the phenomena they represent, indicators do sacrifice a considerable amount of information in the process. The advantage of these measures is obvious, especially when it comes to communicating with stakeholders and focusing attention on specific aspects of complex issues. The disadvantage is the ever-present danger of reducing complexity too much, of flattening out, of losing details and nuances, which are sometimes decisive.

“Indicators arise from values (we measure what we care about), and they create values (we care about what we measure). When […] poorly chosen, they can cause serious malfunctions. Indicators are often poorly chosen”. (Meadows 1998). To avoid pitfalls, an initial point to consider is the limit of the significance of the indicators we construct, i.e., defining what they can accurately, reliably, and correctly represent. Indicators do not have an unlimited semantic carrying capacity. If too many phenomena are crammed into the semantic content of an indicator, the overload results in ambiguous messages that are difficult to interpret and unreliable measurements. Therefore, indicators must be designed in ways that emphasize the logical link between the measured phenomena and the concept that they are supposed to represent as their proxy. This is particularly true in a complex and multi-dimensional field such as Cultural Welfare.

The phenomena we use to build indicators should be dynamic and sensitive enough to allow us to record changes—usually on a yearly basis—in correspondence with interventions or variations in their context. Let us assume that we need to assess the contribution of the visual arts in terms of improving the quality of patients’ stay in hospitals. A first option would be an indicator that measures the “Percentage of hospitals that have commissioned artistic decorations of their wards in the last year”. This is an *input* indicator, and it quantifies the effort, rather than the result (results are measured by *outcome* indicators). It is likely to remain stationary for a long time, as the potential increase in the number of local hospitals that promote artistic decoration tends to be marginal. An alternative measure could be “Percentage of patients in local hospitals who found that decorated wards had a positive effect on their mood”. Such an indicator focuses on the results, involves a larger number of units, and may show variations over time, and possibly across age groups, genders, nationalities, wards, hospitals, etc.

For its composite and complex nature, the *culture and wellbeing* field is remarkably vast and comprehensive. For this reason, I will focus on two purposes of evidence generation in the context of local policies for Cultural Welfare:

i. Surveying and assessment of the level of well-being and health associated with different levels of cultural participation of individuals and groups, also taking into consideration any inequalities in terms of opportunity and any impediments to accessing goods and services related to the arts and culture.
ii. Identifying, monitoring, and evaluating the impact that practiced and experienced cultural activities may have on both individuals and groups.

In the first case, a combination of *supply and demand indicators* shape evidence for policies aimed at reducing inequalities and tailoring opportunities for cultural and artistic practice and participation to the needs of specific groups (e.g., low-income people, the elderly, NEETs, migrants, etc.), also with targeted interventions on local services and facilities.

*Supply indicators* associated with this perspective describe differences in the availability of goods and services, in terms of territorial distribution, quality, variety, etc.

*Demand indicators* quantify access, frequency of experience, perception, practice, and their reported impact on different levels of health and wellbeing. Indicators of this sort may be used in support of urban policy goal setting and goal attainment evaluation. Excessive fragmentation should be avoided. While cultural indicators used in economic assessment tend to measure the intensity of consumption of separate cultural goods and services, like cinemas, theatres, museums, books, magazines, radio, tv, etc., in the context of cultural welfare policies, the focus of measurement should rather be on the contribution that the different cultural experiences may bring to the general wellbeing in an urban/local and everyday dimension. The pioneering study by the University of Umeå (Bygren et al. 1996), showed that the overall cultural and artistic *diet* of people throughout their lives is what impacts their longevity and quality of life the most. Indicators of this kind should capture, on the supply side, the opportunities for an artistic and cultural experience in the selected urban or sub-urban dimension, i.e., the operational daily setting (behaviour setting) of people living in it.

On the *demand side*, indicators should grasp the intensity, variety, and continuity of people’s artistic and cultural activities and experiences over a given period.

On the *supply side*, to assess the differences in the amount, distribution, and variety of public cultural and artistic facilities in different city zones, a possible indicator could be: “varied offer of public cultural and artistic facilities (museums, libraries, theatres, cinemas, concert halls, galleries, etc.) by city district or neighborhood, every 100,000 residents” (UNESCO 2019).

On the *demand side*, a possible indicator could be “Average number of hours per week spent in cultural and artistic activities, including informal—singing, dancing, painting, playing music, listening to music, reading books, visiting museums or galleries, watching theatre performances, etc.—perceived as pleasant and mood-lifting, by city residents by district or neighborhood, gender, and age”.

Data, in this case, are necessarily obtained from surveys and could profit from the integration of objective descriptions of behaviours, like time-budgets, self-reported attendance, automatic or digital counting of admissions and exits, etc., and subjective descriptions of attitudes and feelings (Istat 2019, 2021). This requires investing significant resources, not just for carrying out, but also for re-thinking targeted research. This complex issue is discussed, and recommendations are put forward in a recent Policy Brief submitted to the European Commission (SoPHIA 2021).

The second direction investigates how specific individuals or groups may respond to specific and targeted cultural and artistic activities, specially designed for their health and wellbeing.

Indicators of this kind are used by cultural and artistic organizations when designing their activities aimed at promoting wellbeing and health, and when assessing their impact on different beneficiaries, as well as on their practitioners and other stakeholders. In this sense, indicators provide evidence of the effectiveness of dedicated cultural and artistic practices in bringing about planned and sought-after benefits for selected recipients. About museums, an interesting example is a toolkit devised by researchers at the University College of London and the Arts and Humanities Research Council: “a set of scales of measurement used to assess levels of wellbeing arising from participation in museum and gallery activities that have been trialed across the UK. The Toolkit has been designed to help people involved in running in-house or outreach museum projects, evaluate the impact of this work on the psychological wellbeing of their audiences. The Toolkit is flexible in its application and supports a ‘pick and mix’ approach. It can be used to evaluate the impact of a one-off activity or programme of events” (Thomson and Chatterjee 2013).

In both cases, as in other comprehensive wellbeing measures, cultural welfare indicators should describe “wellbeing outcomes, as opposed to wellbeing drivers measured by input or output indicators. Outcomes may be imperfectly correlated with inputs (e.g., health expenditure may be a poor predictor of health status if the health care system is inefficient) or outputs (e.g., the number of surgical interventions performed may say little about people’s health conditions)” (OECD 2013).

Table 1 proposes examples of indicators that could be used at the local/urban scale for orienting cultural welfare measures.

**Table 1:** Input and outcome indicators of cultural welfare

Purpose	Input	Outcome
Assessment of level of well-being and health associated with different levels of cultural vivacity of individuals and groups, inequalities in terms of opportunity and any impediments to access goods and services related to the arts and culture	(OBJECTIVE INDICATOR, supply). Range of public cultural and artistic venues (museums, libraries, theatres, cinemas, concert halls, galleries, etc.) by city district or neighborhood, every 100,000 residents	(SUBJECTIVE INDICATOR, demand/impact). Average number of hours per week spent engaging in cultural and artistic activities, including informal—singing, dancing, painting, playing music, listening to music, reading books, visiting museums or galleries, watching theatre performances, etc.—perceived as pleasant and mood-lifting, by city residents by district or neighborhood, gender, and age
	(OBJECTIVE INDICATOR, supply). Total amount of integrated artistic and cultural offer (e.g.net hours; net performance units, etc.), per year and territorial area	(SUBJECTIVE INDICATOR, demand/impact). Overall satisfaction with life of people with varying types and levels of cultural and artistic activity: percentage by age, gender, territory
Identifying, monitoring, and evaluating the impact that practiced and experienced cultural activities may have on both individuals and groups	(OBJECTIVE INDICATOR, supply). Percentage of local hospitals that have commissioned artistic decorations of their wards in the last year	(SUBJECTIVE INDICATOR, impact). Percentage of patients in local hospitals who found that decorated wards had a positive effect on their mood
	(OBJECTIVE INDICATOR, supply). Percentage of local museums that have promoted cultural welfare projects in the last year by beneficiary type, number, and duration	(SUBJECTIVE INDICATOR, impact). Percentage of participants in projects of cultural welfare promoted by local museums in the last year who report benefits by beneficiary type, age, gender, and type of benefit

Table 1 combines objective indicators of supply and subjective demand and impact indicators. As suggested earlier in this section, the focus of measurement is both on cultural goods and services with a potential impact on wellbeing made available and on the subjective impact that the various publics report on their wellbeing.

### Time horizons: short, medium, and long term

Cultural welfare indicators may address the impact of the arts and culture on wellbeing at diverse time horizons. Long-term horizons are most common in longitudinal studies, while cross-sectional studies compare different population groups at a single point in time. The first Swedish longitudinal study (Bygren et al. 1996) observed a simple random sample of 15,198 individuals aged 16–74 years. Of these, 85% (12,982) were interviewed between 1982 and 1983 about cultural activities. They were followed up with respect to survival until 31 December 1991.

Another 14 year Swedish study (Konlaan et al. 2000) interviewed 10,609 individuals in 1982 and 1983. The outcome measure was survival until 31st December 1996. In other cases, indicators are tailored to targeted cultural programmes and adopt a medium-term timeframe (Fontanesi and De Souza 2021 on dance and people with Parkinson’s; Bucci et al. 2015 on museums and people with Alzheimer’s), while others measure individual cultural activities immediately after their completion (Clow and Fredhoi 2006 on visiting art galleries; Thomson and Chatterjee 2013 on visiting museums).

### Scale, or the appropriate dimension

So far, while measures for assessing the relationship between culture and wellbeing have been proposed at the national level or at the level of individual organizations or programmes, the level of local/urban policies appears practically unexplored.

The territorial scale of indicators is not a given, although most of the available statistics tend to polarize between the nationwide approach and individual organization focus. Indeed, quantitative measures at the national or sub-national (regional or NUTS III) scale may help comparisons based on rankings. However, when it comes to practical policy choices, indicators tailored to the urban and sub-urban or micro scale are of primary importance, although data collection is costly and often unfeasible due to the insufficient statistical capacity of the local administrations. The basic and regular experiences of culture and the arts which are relevant to the wellbeing and health of individuals, families, and groups take place with diverse frequencies and intensities in their daily *milieux*, made of a plurality of places and activities reachable within 1-h isochrone (Archibugi 1983; Cicerchia 1996). Reading or writing novels, singing in a choir, listening to music, playing in a marching band or simply among friends, watching movies, decorating the house, dancing, drawing, and strolling in an area with nice buildings or beautiful monuments, are normal artistic and cultural experiences that enhance the mood, develop abilities and competences, build, and strengthen social capital and networks. Different places diverge in terms of the intensity, quality, variety, and accessibility of the opportunities and artistic experiences they offer to their residents: some are rich and vibrant, and some are poor and inert. Those experiences develop into habits and shape different profiles of cultural activity and vibrancy that reveal social differences and inequalities (Bourdieu 1984; Levine 1988; Istat 2017). Sure enough, the occasional contact with extraordinary manifestations of art and beauty can trigger violent passions, which can be measured by changes in cortisol and adrenaline levels and can culminate dramatically in Stendhal syndrome—the psychosomatic response experienced while facing aesthetic artistic beauty (Guerrero et al. 2010), but that falls outside the scope of the present exercise. Our focus here is the practical opportunity for sustained access to cultural and artistic experiences offered by territories at the scale of everyday life, as well as the set of policies that help reduce access-related inequalities and break barriers.

The national scale and the sub-national (regional) scale are unable to grasp and render such dynamics. Therefore, official urban and sub-urban delimitations (districts, neighborhoods, etc.), when available, represent a preferable alternative. Indeed, a functional, empirical micro-scale, built on empirical patterns of use rather than fixed administrative or geographical delimitations, would be ideal, although costly and challenging (SoPHIA 2021).

Several practices and studies have selected the community as their preferred local scale for measuring wellbeing—often only with a very tenuous reference to the contribution of culture and the arts. The literature is rich (Pope 2021; Co-Op 2020; Bagnall et al. 2017; Camic 2015; Lee and Kim 2015; Morton and Edwards 2013); its investigation, however, goes beyond the scope of the present exercise. I will simply observe that the notion of community, although fascinating, is blurred and open, changing, and complex, so that specifications are required to operationalize it. “Community can refer to a geographical location such as a neighborhood, village, town, or city; a group of people with shared interests or values; but also, increasingly, to a virtual community based on shared interests, problems, or activities. The term also refers to a participatory process, which encourages collaboration between community members, artists, and others” (Camic 2015). To ensure comparisons and generalisations, fine-grained detail should be counterbalanced by an adequate level of standardisation. This might be hard to attain in practice with the concept of community, whose contents are largely non-territorial.

Another option is to resort to *empirically detected functional areas*, to reconstruct the behavior settings where people have those ordinary—and often informal—experiences of art and culture that create habits, tastes, relations, knowledge, and skills. The Functional Economic Areas in the United States (Fox and Krishna Kumar 1965), and the Local Commuting Systems in Italy (Istat 2010), just to mention some, are functional ex-post areas based upon the detection of usual paths and routes and used for planning and evaluation purposes. “Behavior settings—in the definition by Fox—are the immediate environments of all human behavior and experience. They are objectively defined, directly observable entities with clear-cut boundaries in space and clear-cut beginnings, durations, and endings in time” (Fox 1985). Behavior settings specifically designed for cultural welfare could be delimited by the places, outside of households, where individuals living in an urban center usually experience culture and art: from schools to cinema, from theatre to museums, from galleries to concert halls, libraries, dancing venues, city squares, etc. Data could be obtained through targeted surveys among the resident citizens, as well as from administrative sources, like registries of visitors, the public, etc. This could help create maps, routes, and itineraries to be used as a basis to analyse inequalities. A somewhat similar project is being carried out since 2016 in the city of Rome, which entails the creation of 29 thematic maps of inequalities (Lelo et al. 2019, 2021). The construction of functional areas of cultural activity and engagement could help develop an integrated vision of the territorial offer of cultural and artistic services, venues, opportunities, and facilities, as well as of the personal experience of individuals.

### Data-driven indicators vs framework-driven data collections

Most of the usual indicators related to cultural and artistic supply and demand are built on data collected for purposes different than describing culture and the arts. Eurostat, for instance, declares that “Culture statistics for the EU are not collected by a single stand-alone survey, but come from different Eurostat data collections”. Short of detailed, targeted, harmonized surveys, the artistic and cultural sector and the related practice and participation are underrepresented by the available data, which lack the necessary level of detail, both in territorial and in disciplinary terms. International measures, devised for being universally applicable, are forced to rely on existing data. On the contrary, in the perspective of the present exercise, indicators should reflect a consistent and relevant frame of reference and represent the most significant phenomena, those best suited to describe cultural welfare. The general idea is to encourage targeted data collection, with the specific purpose of populating indicators of cultural wellbeing and welfare. This entails that data will cover both objective—e.g., provision of services, availability of goods and places, etc.—and subjective aspects—e.g., personal assessment or perception of changes in mood, satisfaction, etc., somehow associated with artistic and cultural activities and experiences. To meet the basic qualitative standards, cultural welfare indicators must guarantee independence, impartiality, and objectivity, high quality of the information used (sources, temporal and spatial coverage, etc.), consistency and transparency.

## Conclusions

Upstream of the creation of a system of indicators, there is long and complex work to be done, which needs to fulfill a few conditions.

First, it is necessary to clarify and make explicit the reason for the indicator system, together with who is the most suitable individual or collective subject to build it and for whom.

The second step is to make the framework explicit. Sometimes, the framework is a plan, a programme, or a policy. In the case at hand, it would be preferable to base the indicators on phenomena the development of which depends directly on the outcome of policies, which are measurable at the local level. To define a system of cultural welfare measures, it is necessary to shape the logical model from the generic quality to be measured to the single indicator that represents it satisfactorily and adequately. The indicators of a system should also be limited in number (principle of parsimony) and complementary to each other, covering all dimensions of the system without overlapping, eliminating inconsistencies and redundancies.

The next steps concern the definition of rules and quality criteria (sources, frequency of updates, territorial coverage, etc.) and the final content of the indicator system.

To support planning and evaluation processes, cultural welfare policies need specific, targeted data and indicators. This requires surpassing the limits of a strictly data-driven approach; in other words, rather than shaping the measures solely upon the existing statistics, it is necessary to invest in new data collections tailored to the theoretical and logical framework of the topics involved. I have proposed to consider outcome indicators together with input indicators and suggested that subjective measures could provide valuable information. I have also argued that the urban dimension is the ideal territory for cultural welfare indicators, possibly with further detail, such as official administrative sub-urban units, or, preferably, empirically defined functional areas (communities, behavior settings, etc.). Sets of input and outcome indicators tailored to the individual cultural or artistic organization could also provide valuable information.

Welfare policies that include a substantial contribution of culture and the arts demand to expand and refine the body of evidence at their disposal to orient choices, allocate resources and evaluate impacts and outcomes. Culture statistics, which have been almost exclusively dominated by the economic focus for a long time, could benefit significantly from investments for addressing new emerging phenomena, like wellbeing as a subjectively reported impact.

## Data Availability

Not applicable.

## References

[CR1] Allen J, Allen M, Clift S, Camic PM (2014). The social determinants of health, empowerment, and participation. Oxford textbook of creative arts, health and wellbeing: international perspectives on practice, policy and research.

[CR2] Antonovsky A (1996). The salutogenic model as a theory to guide health promotion. Health Promot Int.

[CR3] Archibugi F (1983) Per una tassonomia generale della domanda di trasporto nei processi di pianificazione dei trasporti. In: CNR. La ricerca sui trasporti in Italia. Franco Angeli, Milano

[CR4] Ascolani F, Cacovean C, Passaretti A, Portaluri T, Sacco PL, Uboldi S, Zbranca R (2020) Art consumption and well-being during the Covid-19 pandemic. https://art-wellbeing.eu/wpcontent/uploads/2021/02/Research-Art-Well-being-during-Covid-19.pdf

[CR5] Bagnall AM, South J, Mitchell B, Pilkington G, Newton R, Di Martino S (2017) Systematic scoping review of indicators of community wellbeing in the UK. https://whatworkswellbeing.org/wp-content/uploads/2020/02/community-wellbeingindicatorsscoping-review-v1-2-aug2017_0205746100.pdf

[CR6] Barker RG (1968). Ecological psychology: concepts and methods for studying the environment of human behavior.

[CR7] Bauer RA (1966). Social indicators.

[CR8] Belfiore E, Clift S, Camic PM (2015). The arts and healing: the power of an idea. Oxford textbook of creative arts, health and wellbeing: international perspectives on practice, policy and research.

[CR9] Bourdieu P (1984). Distinction: a social critique of the judgement of taste.

[CR10] Brajsa-Zganec A, Merkas M, Sverko I (2011). Quality of life and leisure activities: how do leisure activities contribute to subjective well-being?. Soc Indic Res.

[CR11] Brandling J, House W (2009). Social prescribing in general practice: adding meaning to medicine. Br J Gen Pract.

[CR12] Brundtland G (1987) Report of the World Commission on environment and development: our common future. United Nations General Assembly Document A/42/427

[CR13] Bucci C, Carli Ballola L, Černikienė G, Ganß M, Harkin B-A, Jaseliūnienė A, Karpavičiūtė S, Kastner S, Lachi C, Mei M, Petkutė I (2015) Communicating through the arts: a toolkit. http://www.maaproject.eu/moodle/pluginfile.php/244/mod_label/intro/output-ENG.pdf

[CR14] Bygren LO, Konlaan BB, Johansson SE (1996). Attendance at cultural events, reading books or periodicals, and making music or singing in a choir as determinants for survival: Swedish interview survey of living conditions. BMJ.

[CR15] Camic PM, Clift S, Camic PM (2015). Community cultural development for health and wellbeing. Oxford textbook of creative arts, health and wellbeing: international perspectives on practice, policy and research.

[CR16] Canadian Index of Wellbeing (2016). How are Canadians really doing? The 2016 CIW national report.

[CR17] Carnwath J, Brown AS (2014) Understanding the value and impacts of cultural experiences: a literature review. Arts Council England. https://www.artscouncil.org.uk/publication/understanding-value-andimpacts-culturalexperiences

[CR18] Chadha N, Coticchia B, Harpreet G, Pokharna R, Posadas F, Pereira E, Richter P, Stopak-Behr Z (2015) State of New Yorkers—a well-being index. https://www1.nyc.gov/assets/cidi/downloads/pdfs/nyc_well_being_index_full_report_2015.pdf

[CR19] Cicerchia A (1996). Indicators for the measurement of the quality of urban life. Soc Indic Res.

[CR20] Cicerchia A (2019). Evidence-based policy making for cultural heritage. SCIRES-IT.

[CR22] Cicerchia A (2021) Che cosa muove la cultura. Impatti, misure e racconti tra economia e immaginario. Bibliografica, Milano

[CR21] Cicerchia A, Rossi Ghiglione A, Seia C (2020) Welfare culturale. Atlante della cultura Treccani, Roma

[CR23] Clair AA (1996). Therapeutic uses of music with older adults.

[CR24] Clift S, Camic PM (2015). Oxford textbook of creative arts, health and wellbeing: international perspectives on practice, policy and research.

[CR25] Clift S, Hancox G (2010). The significance of choral singing for sustaining psychological wellbeing: findings from a survey of choristers in England, Australia and Germany. Music Perform Res.

[CR26] Clift S, Hancox G, Staricoff R, Whitmore C (2008). Singing and health: a systematic mapping and review of non-clinical research.

[CR27] Clift S, Phillips K, Pritchard S (2021). The need for robust critique of research on social and health impacts of the arts. Cultural Trends.

[CR28] Clow A, Fredhoi C (2006). Normalisation of salivary cortisol levels and self-report stress by a brief lunchtime visit to an art gallery by London City workers. J Holist Healthc.

[CR29] Cobb C, Halstead T, Rowe J (1995). The genuine progress indicator, summary of data and methodology.

[CR30] Cohen JL, Johnson L, Orr P (2016). Video and filmmaking as psychotherapy. Research and practice.

[CR31] Co-Op (2020) The community wellbeing index October 2020 update. https://communitywellbeing.coop.co.uk/media/1041/cwi_technical_report-2021.pdf

[CR32] Copeland R, Cohen M (1983). What is dance? Readings in theory and criticism.

[CR33] Cork R (2012). The healing presence of art.

[CR34] Cuyper K, Krokstad S, Lingaas Holmen T, SkjeiKnudtsen M, OlovBygren L, Holmen J (2011). Patterns of receptive and creative cultural activities and their association with perceived health, anxiety, depression and satisfaction with life among adults: the HUNT study, Norway. J Epidemiol Community Health.

[CR35] Dalziel P, Saunders C, Savage C (2019) Culture, wellbeing, and the living standards framework: a perspective. Discussion paper 19/02—prepared for the Ministry for Culture and Heritage and the Treasury. New Zealand Treasury, Wellington

[CR36] Dermer SB, Hutchings JB (2010). Utilizing movies in family therapy: applications for individuals, couples, and families. Am J Fam Ther.

[CR37] Desmarais S, Bedford L, Chatterjee HJ (2018) Museums as spaces for wellbeing: a second report from the National Alliance for Museums, Health and Wellbeing. www.museumsandwellbeingalliance.wordpress.com

[CR38] Diener E, Suh E (1997). Measuring quality of life: economic, social, and subjective indicators. Soc Indic Res.

[CR39] Durdey T (2006) Advance: creating wellbeing through movement and dance, Dance 4

[CR40] Eurostat (2010) GDP and beyond. Sygma—The Bulletin of European Statistics 02 2010

[CR41] Fancourt D, Finn S (2019) Health evidence network synthesis report 67. What is the evidence on the role of the arts in improving health and well-being? A scoping review. WHO Regional Office for Europe, Copenhagen 32091683

[CR42] Fears A (2011). The museum as a healing space: addressing museum visitors’ emotional responses through viewing and creating artwork.

[CR43] Fontanesi C, DeSouza JFX (2021). Beauty that moves: dance for Parkinson’s effects on affect, self-efficacy, gait symmetry, and dual task performance. Front Psychol.

[CR44] Fox KA (1985). Social system accounts based on behavior settings: some next steps. Social system accounts. Theory and decision library.

[CR45] Fox KA (1986). The present status of objective social indicators: a review of theory and measurement. Am J Agric Econ.

[CR46] Fox KA, Ghosh S (1981). A behavior setting approach to social accounts combining concepts and data from ecological psychology, economics, and studies of time use. Social accounting systems: essays on the state of the art.

[CR47] Fox KA, Krishna Kumar T (1965). The functional economic area: delineation and implications for economic analysis and policy. Pap Reg Sci.

[CR48] Gaffaney J (2017) Thriving cities: how to define, apply, and measure well-being at scale. Master of applied positive psychology (MAPP) Capstone Projects. 120. https://repository.upenn.edu/mapp_capstone/120

[CR49] Garcia B, Cox T (2013) European capitals of culture: success strategies and long-term effects. study for the European parliament's committee on culture and education. https://www.europarl.europa.eu/RegData/etudes/etudes/join/2013/513985/IPOLCULT_ET%282013%29513985_EN.pdf

[CR50] Grossi E, TavanoBlessi G, Sacco PL (2019). Magic moments: determinants of stress relief and subjective wellbeing from visiting a cultural heritage site. Cult Med Psychiatry.

[CR51] Guerrero AL, Barceló Rosselló A, Ezpeleta D (2010). Síndrome de Stendhal: origen, naturaleza y presentación en un grupo de neurólogos. Neurologia.

[CR52] Gump PV (1971). The behavior setting: a promising unit for environmental designers. Landsc Archit.

[CR53] Helliwell J, Layard R, Sachs J (2012). World happiness report.

[CR54] Hill K (2021) Canadians’ arts participation, health, and well-being. SIA report 53. Hill Strategies Research Inc., Hamilton

[CR55] Hjort M (2019). The public value of film: moving images, health, and well-being. J Scand Cine.

[CR56] Hui A, Stickley T (2010). The elders dance project: a study of a culturally sensitive initiative. Br J Wellbeing.

[CR58] Istat (2010) I Sistemi Locali del Lavoro. Nota Metodologica. https://www.istat.it/it/files//2014/12/nota-metodologica_SLL2011_rev20150205.pdf

[CR59] Istat (2013) Il Benessere Equo e Sostenibile in Italia 2013. Istituto Nazionale di Statistica, Roma

[CR57] Istat (2017) Aspetti demografici e condizioni di vita. In Rapporto 2017. La situazione del Paese. Istituto Nazionale di Statistica, Roma

[CR60] Istat (2019) i tempi della vita quotidiana. Lavoro, conciliazione, parità di genere e benessere soggettivo. Istituto Nazionale di Statistica, Roma

[CR61] Istat (2021) Il Benessere Equo e Sostenibile in Italia 2021. Istituto Nazionale di Statistica, Roma

[CR62] Jackson MR, Kabwasa-Green F, Herranz J (2006). Cultural vitality in communities: interpretation and indicators.

[CR1001] James P (2015). Urban sustainability in theory and practice. Circles of sustainability.

[CR64] Kennedy RF (1968) Remarks at the University of Kansas, March 18, 1968. MR 89-34. Miscellaneous recordings, John F. Kennedy Presidential Library. https://www.jfklibrary.org/learn/about-jfk/the-kennedy-family/robert-f-kennedy/robert-fkennedy-speeches/remarks-at-the-university-of-kansas-march-18-1968

[CR65] Konlaan BB, Bygren LO, Johansson SE (2000). Visiting the cinema, concerts, museums or art exhibitions as determinant of survival: a Swedish fourteen-year cohort follow-up. Scand J Public Health.

[CR66] Kuznets S (1934) National Income, 1929–1932. A report on national income, 1929–32 January 4, 1934. Referred to the Committee on Finance. United States Government Printing Office, Washington DC, p 7

[CR68] Land K (1983). Social indicators. Ann Rev Sociol.

[CR69] Lee SJ, Kim Y, Kim Y, Phillips R (2015). Searching for the meaning of community well-being. Community well-being and community development.

[CR70] Lelo K, Monni S, Tomassi F (2019) Le mappe della disuguaglianza. Una geografia sociale metropolitana. Donzelli, Roma

[CR71] Lelo K, Monni S, Tomassi F (2021) Le sette Rome. La capitale delle disuguaglianze raccontate in 29 mappe. Donzelli, Roma

[CR72] Levine LW (1988). Highbrow/lowbrow: the emergence of cultural hierarchy in America.

[CR73] Lewin K (1951). Field theory in social science.

[CR75] Matthew A, Lenahan P, Pavlov A (2012). Cinemeducation: using film and other visual media in graduate and medical education.

[CR76] McLellan R, Galton M, Steward S, Page C (2012). The impact of creative initiatives on wellbeing: a literature review.

[CR77] Meadows DH (1998). Indicators and information systems for sustainable development.

[CR78] Meeks S, Vandenbroucke RJ, Shryock SK (2020). Psychological benefits of attending the theatre associated with positive affect and wellbeing for subscribers over age 60. Aging Ment Health.

[CR79] Michalak EE, Livingston JD, Maxwell V, Hole R, Hawke LD, Parikh SV (2014). Using theatre to address mental illness stigma: a knowledge translation study in bipolar disorder. Int J Bipolar Disord.

[CR80] Miringoff ML (1997). The index of social health 1996: monitoring the social well-being of the nation.

[CR81] Mirza M (ed) (2006) Culture vultures: is UK arts policy damaging the arts? Policy Exchange. https://www.policyexchange.org.uk/wp-content/uploads/2016/09/culture-vultures-jan-06.pdf

[CR82] Montalto V, Tacao Moura C, Panella F, Alberti V, Becker W, Saisana M (2019). The cultural and creative cities monitor: 2019 edition.

[CR83] Morton A, Edwards L (2013) Community wellbeing indicators. Measures for local government. Survey template for local government, Australian Centre of Excellence for Local Government, University of Technology, Sydney

[CR84] Noble G, Chatterjee H (2013). Museums, health and well-being.

[CR85] Noll H-H, Zapf W, Borg I, Ph Mohler P (1994). Social indicators research: societal monitoring and social reporting. Trends and perspectives in empirical social research.

[CR86] Nordhaus WD, Tobin J, Moss M (1973). Is growth obsolete?. The measurement of economic and social performance.

[CR87] OECD (1982). The OECD list of social indicators.

[CR600] OECD (2011) How's Life?: Measuring Well-being, OECD Publishing, Paris. 10.1787/9789264121164-en

[CR88] OECD (2013). How’s life? Measuring well-being 2013.

[CR89] OECD (2021) Economic and social impact of cultural and creative sectors. Note for Italy G20 Presidency Culture Working Group. https://www.oecd.org/cfe/leed/OECD-G20-Culture-July2021.pdf

[CR90] Orii L, Alonso L, Larson K (2020). Methodology for establishing well-being urban indicators at the district level to be used on the CityScope platform. Sustainability.

[CR91] Owen JW (ed) (2013) Arts, health and wellbeing beyond the millennium: how far have we come and where do we want to go? p 4. https://www.rsph.org.uk/our-work/policy/wellbeing/arts-andhealth.html

[CR92] Pineo H, Rydin Y (2018). Cities, health, and well-being.

[CR93] Pope J (2021). Indicators of community wellbeing for the southern and hills local government area.

[CR94] Powell ML, Newgent RA (2010). Improving the empirical credibility of cinematherapy: a single-subject interrupted time-series design. Couns Outcome Res Eval.

[CR96] Royal Government of Bhutan (2012) The report of the high-level meeting on wellbeing and happiness: defining a new economic paradigm. Office of the Prime Minister, Thimphu

[CR99] Sacco PL (2017a) Appunti per una definizione di welfare culturale. Giornale delle Fondazioni. http://www.ilgiornaledellefondazioni.com/content/appunti-una-definizione-diwelfare-culturale-1

[CR97] Sacco PL (2017). Health and cultural welfare: a new policy perspective?. Econ Cult.

[CR98] Sacco PL, Grossi E (2015) Impact of culture on individual well-being. PPP Ispra 11 June 2015

[CR100] Sartorius N (2006). The meanings of health and its promotion. Croat Med J.

[CR101] Sextou P, Monk C (2013). Bedside theatre performance and its effects on hospitalised children’s wellbeing. Arts Health.

[CR102] Sigurdson O (2015). Culture and health. A wider horizon.

[CR103] Smith R (2002). Spend (slightly) less on health and more on the arts—health would probably be improved. BMJ.

[CR104] SoPHIA—Social Platform for Holistic Heritage Impact Assessment Consortium (2021) Deliverable D 3.5: policy brief with recommendations on economic impact for policy makers. https://sophiaplatform.eu/en/archive

[CR105] Sterling P (2019). What is health? Allostasis and the evolution of human design.

[CR106] Stiglitz J, Sen A, Fitoussi J-P (2009) Report by the commission on the measurement of economic performance and social progress, Paris. www.Stiglitz-Sen-Fitoussi.fr

[CR107] The Beaney House of Art and Knowledge (2021) The Beaney health and wellbeing in museums toolkit. Canterbury Museums and Galleries, Canterbury

[CR108] The Heritage Alliance (2020) Heritage, health and wellbeing. A heritage alliance report. https://www.theheritagealliance.org.uk/wp-content/uploads/2020/10/Heritage-AllianceAnnualReport_2020_Online.pdf

[CR109] The Wellbeing Project (2017) Creating a city for wellbeing: key findings about wellbeing perspectives and assets in Santa Monica Wave 2/Version 2.0. PPP. https://santamonicawellbeing.org/Media/Default/docs/CSM_wellbeing_briefing_deck_2017_FINAL.pdf

[CR110] Thomson J, Chatterjee HJ (2013) UCL museum wellbeing measures toolkit. p 3. https://www.ucl.ac.uk/culture/sites/culture/files/ucl_museum_wellbeing_measures_toolkit_sept2013.pdf

[CR111] UNDP—United Nations Development Program (1994) Human development report 1994: new dimensions of human security. http://www.hdr.undp.org/en/content/human-developmentreport-1994

[CR113] UNESCO (2015) UNESCO’s work on culture and sustainable development: evaluation of a policy theme. IOS/EVS/PI/145 REV.5. UNESCO, Paris, p 62

[CR112] UNESCO (2019). Culture 2030 indicators.

[CR114] United Nations General Assembly (2012) Resolution adopted by the General Assembly on 28 June 2012. International Day of Happiness. (A/66/L.48/Rev.1) 66/281. The United Nations, New York

[CR115] US Bureau of Labour Statistics (2021) ATUS well-being module questionnaire. https://www.bls.gov/tus/wbmquestionnaire.pdf

[CR116] US Department of Health, Education and Welfare (Hew) (1969) Toward a social report. US Government Printing Office, Washington, DC

[CR117] Veall D (2017). Museums on prescription: a guide to working with older people.

[CR119] Wilkinson AV, Waters AJ, Bygren LO, Tarlov AR (2007). Are variations in rates of attending cultural activities associated with population health in the United States?. BMC Public Health.

[CR120] World Health Organization (1948). Constitution of the World Health Organization.

[CR122] World Health Organization Commission on Social Determinants of Health (2008). Closing the gap in a generation. Health equity through action on the social determinants of health.

